# Prognostic Value of Liver Stiffness in Advanced Chronic Liver Disease After Hepatitis C Virus Eradication

**DOI:** 10.1111/liv.70869

**Published:** 2026-09-15

**Authors:** Federica Cerini, Mariagrazia Rumi, Jonas Schropp, Agostino Cosenza, Carmelo Selvaggio, Chiara Masellis, Annalisa Berzigotti

**Affiliations:** ^1^ Hepatology Unit San Giuseppe Hospital, University of Milan Milan Italy; ^2^ Department of Clinical Sciences and Community Health University of Milan Milan Italy; ^3^ Department for Visceral Medicine and Surgery, Inselspital Bern University Hospital, University of Bern Bern Switzerland; ^4^ Department of Biomedical Research University of Bern Bern Switzerland; ^5^ Bavarian Center for Digital Health and Social Care Kempten University of Applied Sciences Kempten Germany; ^6^ Graduate School for Health Sciences University of Bern Bern Switzerland

**Keywords:** advanced chronic liver disease, liver cirrhosis, liver related events, liver stiffness measurement

## Abstract

**Background & Aim:**

Sustained‐virological‐response (SVR) after direct‐acting‐antiviral (DAA) therapy improves outcomes in patients with HCV‐related compensated‐advanced‐chronic‐liver‐disease (cACLD). However, it remains unclear whether absolute post‐SVR LSM or LSM reduction better predicts prognosis after SVR. This study evaluated the prognostic performance of absolute‐values‐LSM versus dynamic‐change for predicting liver‐related‐events after SVR.

**Methods:**

We retrospectively analysed patients with HCV‐related cACLD treated with DAA between December 2014 and March 2022. Baseline and post‐SVR non‐invasive tests (LSM, FIB‐4 and APRI) were assessed. Predictive performance for liver‐related events was evaluated using time‐dependent receiver operating characteristic analysis. Associations between Baveno‐VII criteria for clinically significant LSM improvement and clinical outcomes were assessed using Fine–Grey competing‐risk models.

**Results:**

During a median of 60 months (95%; CI 52–69), 11/135 patients (8%) developed liver‐related events. Patients who developed events had higher baseline LSM than those who remained event‐free (24 vs. 18 kPa, *p* = 0.049), and higher follow‐up LSM values (20 vs. 12 kPa, *p* = 0.043). Follow‐up LSM showed good discriminative ability for liver‐related events within the first 3 years after SVR (tAUC at 2 years: 0.86 [95% CI 0.75–0.97]; tAUC at 3 years: 0.82 [0.71–0.93]). Follow‐up LSM was significantly superior to ΔLSM and percentage ΔLSM at both 2 and 3 years (all *p* ≤ 0.028). In competing‐risk analysis, only follow‐up LSM > 20 kPa was significantly associated with liver‐related events (subdistribution hazard ratio 5.61, *p* = 0.007), whereas Baveno‐VII criteria for LSM regression were not (SHR 0.33, *p* = 0.097).

**Conclusions:**

Absolute post‐treatment‐LSM provides stronger prognostic information than dynamic changes from baseline. These findings support repeated post‐SVR LSM assessment for risk stratification suggesting the relevance of residual liver disease in predicting liver‐related outcomes.

## Lay Summary

- Achieving a sustained virological response (SVR) in patients with advanced chronic liver disease (ACLD) due to HCV can lead to better health outcomes, in particular in the presence of significant LSM improvement after treatment.
- This study examined whether the change in liver stiffness (from before to after treatment) or the absolute liver stiffness measurement after treatment is more useful in predicting liver complications over time in patients who have achieved SVR.
- The study found that the absolute value of liver stiffness measured after treatment (FU‐LSM) was the best predictor for liver‐related events. Changes in liver stiffness from before treatment did not reliably predict these complications, supporting the use of absolute liver stiffness values during follow‐up for monitoring.

## Key Points

- SVR improves outcomes in patients with HCV‐related advanced chronic liver disease (ACLD).
- Baseline and post‐treatment LSM (FU‐LSM) showed good predictive ability for hepatic decompensation at 2 and 3 years after SVR. FIB‐4 and APRI also showed good predictive ability for decompensation during the first 36 months after SVR.
- Absolute LSM during follow‐up is the strongest prognostic indicator of liver‐related events.
- Post‐SVR absolute LSM, particularly FU‐LSM > 20 kPa, is a stronger predictor of hepatic decompensation than the relative change in LSM from baseline.

**Abbreviations**
BL: baseline
cACLD: compensated advanced chronic liver disease
CSPH: clinically significant portal hypertension
DAAs: direct‐acting antivirals
EoT: end‐of‐treatment
FU: follow‐up
HCC: hepatocellular carcinoma
LSM: liver stiffness measurement
MI: multiple imputation
PLT: platelet count
SHR: subdistribution hazard ratio
SVR: sustained virological response
tAUC: Area under the time‐dependent receiver‐operating characteristics curve

## 1 Introduction

Direct‐acting antiviral (DAA) therapies have revolutionized the treatment of liver disease due to Hepatitis C Virus (HCV), offering cure rates exceeding 95% in most patients. Achieving sustained virological response (SVR)—the absence of detectable HCV RNA 12 weeks after completing treatment—is associated with a range of positive outcomes, including liver health improvement [1, 2, 3, 4].

Liver stiffness measurement (LSM) is a well‐established non‐invasive surrogate of liver fibrosis in patients with HCV [5, 6]. Beyond staging of fibrosis, LSM is a key component of the non‐invasive diagnosis of clinically significant portal hypertension (CSPH) [7], and it allows stratifying the risk of liver‐related events in this population, and in compensated advanced chronic liver disease (ACLD) in general [7, 8, 9, 10].

The Baveno VII Consensus Workshop suggested using a pragmatic “rule‐of‐5” based on LSM to rule‐out and rule‐in cACLD and CSPH and the progressively higher relative risks of decompensation and liver‐related death independently of the aetiology [11, 12].

However, ACLD is a highly dynamic condition which can transition not only from the compensated to the decompensated stage, but also in the opposite direction.

Favourable outcomes have been reported among patients with compensated disease who achieve a liver stiffness measurement (LSM) below 20 kPa. Similarly, a significant reduction in LSM over time has also been associated with improved prognosis, particularly in patients demonstrating a decrease of at least 20% from baseline and/or reaching an LSM value below 10 kPa [7, 12].

However, this criterion still requires validation in independent cohorts, particularly regarding the best prognostic stratification following SVR either based on absolute post‐treatment LSM values or longitudinal changes in LSM.

In the present work we aimed to evaluate the prognostic performance of absolute values of LSM versus their change for liver‐related events during follow‐up after viral eradication, in patients with chronic liver disease due to HCV infection.

## 2 Methods

### 2.1 Study Design and Patients

This retrospective longitudinal cohort study was conducted at the Hepatology Unit of San Giuseppe Hospital, University of Milan. We screened consecutive patients with compensated advanced chronic liver disease (cACLD) due to chronic hepatitis C virus (HCV) infection who achieved sustained virological response (SVR) after interferon‐free direct‐acting antiviral (DAA) therapy between December 2014 and March 2022.

Patients were eligible if they had available LSM and laboratory data at baseline and at least one post‐SVR follow‐up assessment. Follow‐up continued until February 2024.

Patients with partially missing follow‐up data were included in the analysis and missing values were handled using multiple imputation.

The study included individuals who met the following criteria: age of at least 18 years old, compensated chronic liver disease due to a history of HCV infection, treatment with interferon‐free direct‐acting antiviral (DAA) regimens, availability of LSM and laboratory data at baseline and at least one post‐SVR evaluation.

Patients with missing data on follow‐up were still included in the analysis and evaluated by multiple imputation (MI) to fill the missing information. A flow diagram for study participants was shown in Figure S1.

Exclusion criteria were pregnancy or breastfeeding, coinfection with HIV, other chronic liver diseases (e.g., autoimmune hepatitis, primary biliary cholangitis, primary sclerosing cholangitis, hemochromatosis, Wilson's disease), prior liver transplantation, prior tranjugular intrahepatic portosystemic shunt, previous liver related events and missing data on both LSM and laboratory.

A flow diagram for study participants was shown in Figure S1.

### 2.2 Liver Stiffness Measurement (LSM)

All LSM assessments were performed by experienced operators using vibration‐controlled transient elastography (FibroScan compact 530; Echosens, Paris, France), adhering to the local standard operating procedure and established quality criteria [13, 14].

Patients were fasting for at least 3 h prior to the procedure and were lying in a supine position, with the right arm raised above the head to facilitate access to the liver. Measurements were performed in the right lobe of the liver through intercostal spaces, and the M and XL probes were chosen based on the probe selection tool.

Results were considered reliable, in case of at least 10 valid measurements with an interquartile range (IQR) less than 30% of the median stiffness value.

### 2.3 Data Collection and Definitions

Demographic characteristics and data on BMI, diabetes, components of the metabolic syndrome and alcohol consumption were collected from patients' records, together with laboratory parameters and LSM before and after HCV cure. Due to the retrospective design of this study, the timepoints of post‐SVR assessment were not standardized.

Data on the clinical course during follow‐up were collected from chart review starting from the EoT until February 2024. Liver related events were defined as the occurrence of variceal bleeding or development of clinically overt ascites (grade 2 or 3), overt hepatic encephalopathy (West Haven grade ≥ 2) or portal vein thrombosis [15].

Hepatocellular carcinoma (HCC) was diagnosed according to clinical practice guidelines of the European Association for the Study of the Liver, and was considered a relevant liver‐related event [16, 17].

For outcome analyses, follow‐up started at the time of post‐SVR assessment.

According to Baveno VII, patients with ACLD have to be periodically monitored with LSM to identify any change in LSM value which is associated with a reduction in risk of liver‐related events and death, defined as a decrease in LSM ≥ 20% associated with LSM < 20 kPa or any decrease to a LSM < 10 kPa [12]. In this study changes in LSM were calculated as below: Baseline LSM is the LSM at the beginning of treatment; follow‐up LSM (FU‐LSM) is the first LSM post‐SVR; Delta‐LSM is the change in LSM between baseline and first follow‐up (Baseline LSM—FU‐LSM); Delta‐LSM in percent is Delta‐LSM as a percentage of baseline LSM ((Baseline LSM—FU‐LSM)/(Baseline LSM) * 100); Delta‐LSM per month is the rate of change in LSM over time between baseline and FU (Baseline LSM—FU‐LSM)/(time between baseline and 1st FU in months).

FU‐LSM was assessed approximately 12 months after SVR. FIB‐4 and APRI were measured at the same time point.

The study was conducted in accordance with the Declaration of Helsinki and was approved by the local ethics committee (Study number 4288 ID study 4658).

### 2.4 Objectives

The aim of the study is to evaluate the prognostic performance of absolute values of LSM versus their change over the course of DAA treatment for liver‐related events.

The primary outcome was to evaluate the association between longitudinal changes in LSM and the occurrence of liver‐related events in patients with advanced chronic liver disease (ACLD). As a secondary outcome, we compared the abilities of dynamic changes in liver stiffness measurement (LSM) and absolute LSM values to predict liver‐related events.

Another goal was to assess the relationship between LSM changes and liver‐related mortality. Additionally, the study aimed to validate the Baveno VII definition of a clinically significant decrease in LSM.

### 2.5 Statistical Analysis

Continuous and ordered variables are described as medians with interquartile range, categorical variables as counts and percentages. Descriptive statistics are provided for the overall group and stratified by whether patients experienced any liver‐related event during follow‐up. Groups were compared using the Wilcoxon rank sum test and the chi‐squared test or Fisher's exact test, as appropriate. The same comparisons are provided for baseline, follow‐up, delta and delta as a percent of baseline values for LSM, FIB‐4 and APRI as well as LSM‐based Baveno criteria. We used the same methods for Table S1, which compares baseline variables over whether LSM (kPa) decreased after treatment. For descriptive statistics, missing values were excluded. Cumulative event rates at 1–8 years after the start of treatment were estimated using the Kaplan–Meier method for relevant outcomes.

The area under the time‐dependent receiver operating curve (time‐AUC) at 2, 3, 4 and 5 years post treatment start is used to quantify the predictive accuracy of the non‐invasive tests and their change dynamics. Analyses were conducted both as complete‐case analyses, restricted to patients with the measurements required for the respective predictor, and after multiple imputation. Fifty imputed datasets were generated by chained equations using the MICE algorithm. The imputation model included event indicators, log‐transformed event times and their interactions, baseline laboratory values, patient characteristics, NITs and disease‐severity scores.

For each imputed dataset, AUCs were logit‐transformed, and their standard errors were estimated using influence‐function representations [18]. Estimates were combined using Rubin's rules [19] and subsequently back‐transformed to the original scale to obtain pooled AUCs and 95% confidence intervals. Pairwise differences between AUCs were evaluated using influence‐function‐based covariance estimates. Results were pooled across imputed datasets using Rubin's rules, and *p*‐values were obtained from the corresponding multivariate normal distribution while accounting for comparisons across time points. Given the large number of exploratory comparisons, these *p*‐values should be interpreted cautiously.

Associations between the Baveno criteria and liver‐related events were evaluated using Fine–Grey competing‐risk regression, treating death as a competing event. These models were fitted in both the complete‐case and multiply imputed datasets.

All statistical analyses were done with R version 4.3.2. The cutoff for statistical significance is set at a two‐tailed *p*‐value of < 0.05.

## 3 Results

### 3.1 Baseline Characteristics

Of 156 patients with cACLD due to HCV infection treated with direct‐acting antivirals, 135 were included in the analysis. Twenty‐one patients were excluded because both elastographic and laboratory data were missing at baseline or follow‐up, or for evidence of decompensated liver disease at baseline or during therapy. At SVR, the cohort consisted predominantly of older adults with advanced chronic liver disease, as reflected by elevated baseline LSM values. Approximately half of the patients were overweight. Patients who developed liver‐related events during follow‐up showed worse baseline liver function (lower albumin, higher bilirubin, INR and MELD score), higher baseline LSM and a higher prevalence of significant oesophageal varices compared with those who remained compensated. Non‐invasive serum fibrosis scores were not significantly different between groups.

The main characteristics of the population of the study are summarized in Table 1 and Table 2.

**TABLE 1:** Baseline Patients' characteristics in the overall cohort (*N* = 135).

Characteristic	Missing values	Overall *n* = 135^a^	Liver‐related events *n* = 11^a^	No liver‐related events *n* = 124^a^	*p* ^b^
Male sex	0	75 (56%)	7 (64%)	68 (55%)	0.8
Age (years)	0	69 (55, 76)	74 (62, 81)	68 (55, 75)	0.14
LSM (kPa)	1	18 (15, 23)	24 (18, 33)	18 (15, 23)	0.049
LSM groups (kPa)	1				0.3
< 10		2 (1.5%)	0 (0%)	2 (1.6%)	
10–20		74 (55%)	4 (36%)	70 (57%)	
> 20		58 (43%)	7 (64%)	51 (41%)	
Probe (M/XL)	4	94 (72%)/37 (28%)	8 (73%)/3 (27%)	86 (72%)/34 (28%)	> 0.9
Fib‐4	1	5.0 (2.9, 8.6)	7.8 (4.2, 9.5)	4.9 (2.8, 7.6)	0.10
Fib‐4 > 3.25	1	92 (69%)	10 (91%)	82 (67%)	0.2
APRI	1	1.58 (0.93, 2.86)	2.73 (1.26, 3.29)	1.57 (0.86, 2.81)	0.3
APRI > 2	1	54 (41%)	6 (55%)	48 (39%)	0.4
spleen size (mm)	0	125 (108, 140)	135 (113, 150)	125 (106, 136)	0.2
PV diameter (mm)	2	12.00 (11.00, 13.00)	13.00 (11.50, 14.00)	11.40 (10.25, 13.00)	0.037
presence and grade of varices	17				0.021
0 (includes previous EBL)		90 (76%)	7 (64%)	83 (78%)	
F1		22 (19%)	1 (9.1%)	21 (20%)	
F2		4 (3.4%)	2 (18%)	2 (1.9%)	
F3		2 (1.7%)	1 (9.1%)	1 (0.9%)	
Platelet count (×10^9^/L)	0	129 (87, 169)	99 (83, 131)	134 (91, 169)	0.3
Albumin (g/dL)	0	4.00 (3.73, 4.30)	3.73 (3.46, 3.95)	4.00 (3.77, 4.30)	0.020
Bilirubin (mg/dL)	0	0.86 (0.70, 1.20)	1.34 (0.95, 1.55)	0.81 (0.68, 1.20)	0.016
AST (U/L)	0	73 (50, 126)	84 (56, 116)	73 (50, 127)	0.7
ALT (U/L)	0	83 (53, 115)	89 (53, 100)	83 (54, 120)	0.5
gGT (U/L)	0	72 (45, 127)	82 (58, 133)	71 (45, 121)	0.5
INR	2	1.10 (1.05, 1.20)	1.20 (1.14, 1.28)	1.10 (1.05, 1.19)	0.016
Glycemia (mg/dL)	11	100 (90, 112)	101 (93, 112)	100 (88, 112)	0.7
Total Cholesterol (mg/dL)	23	160 (140, 183)	148 (141, 166)	161 (140, 183)	0.4
Tryglicerides (mg/dL)	29	98 (74, 123)	123 (96, 128)	98 (74, 122)	0.2
aFP (ng/mL)	18	9 (5, 23)	9 (6, 34)	9 (5, 22)	0.5
Creatinine (mg/dL)	10	0.75 (0.65, 0.89)	0.80 (0.66, 0.92)	0.75 (0.66, 0.87)	0.6
Child‐Pugh class (A/B/C)	3	121 (92%)/11 (8.3%)/0 (0%)	9 (82%)/2 (18%)/0 (0%)	112 (93%)/9 (7.4%)/0 (0%)	0.2
MELD	12	8.00 (7.00, 10.00)	10.00 (9.00, 11.75)	8.00 (7.00, 9.00)	0.008
BMI	0	24.9 (23.0, 27.7)	24.8 (23.9, 26.8)	25.0 (22.9, 27.6)	> 0.9
BMI > 25	0	67 (50%)	4 (36%)	63 (51%)	0.4
BMI > 30	0	14 (10%)	0 (0%)	14 (11%)	0.6
DMT2 (yes)	0	32 (24%)	4 (36%)	28 (23%)	0.3
HbA1c (mmol/mol)	112	41 (37, 55)	35 (34, 37)	42 (37, 55)	0.2
Dyslipidemia	30	30 (22%)	3 (27%)	27 (22%)	0.7
Statins	9	9 (6.7%)	1 (9.1%)	8 (6.5%)	0.5
Arterial hypertension	0	68 (50%)	6 (55%)	62 (50%)	0.8
Alcohol consumption (≥ 20 g/day for women; ≥ 30 g/day for men)	0	18 (13%)	0 (0%)	18 (15%)	0.4

Abbreviations: AFP, alpha‐fetoprotein (ng/mL); ALT, alanine aminotransferase (U/L); APRI, aspartate aminotransferase‐to‐platelet ratio index; AST, aspartate aminotransferase (U/L); BMI, body mass index (kg/m^2^); DMT2, type 2 diabetes mellitus; EBL, endoscopic band ligation; FIB‐4, Fibrosis‐4 index; gGT, gamma‐glutamyl transferase (U/L); HbA1c, glycated haemoglobin; INR, international normalized ratio; LSM, liver stiffness measurement (kPa); MELD, Model for End‐Stage Liver Disease score; PV, portal vein diameter (mm).

^a^ *n* (%); Median (IQR).

^b^ Fisher's exact test; Wilcoxon rank sum test; Pearson's Chi‐squared test.

**TABLE 2:** Elastografic assessment and criteria for CSPH regression in the study cohort (*N* = 135) and according to liver‐related events.

Characteristic	Missing values	Overall, *n* = 135^a^	Liver‐related events *n* = 11^a^	No liver‐related events *n* = 124^a^	*p* ^b^
Baseline LSM (kPa)	1	18 (15, 23)	24 (18, 33)	18 (15, 23)	0.049
Baseline LSM (kPa)	1				0.3
< 10		2 (1.5%)	0 (0%)	2 (1.6%)	
10–20		74 (55%)	4 (36%)	70 (57%)	
> 20		58 (43%)	7 (64%)	51 (41%)	
FU‐LSM (kPa)	8	12 (8, 17)	20 (12, 24)	12 (8, 17)	0.043
FU‐LSM (kPa) groups	8				0.028
< 10		49 (39%)	3 (30%)	46 (39%)	
10–20		55 (43%)	2 (20%)	53 (45%)	
> 20		23 (18%)	5 (50%)	18 (15%)	
Delta LSM (kPa)	9	7 (4, 11)	7 (4, 12)	7 (4, 11)	0.7
Delta LSM (%)	9	0.36 (0.22, 0.51)	0.38 (0.15, 0.44)	0.36 (0.22, 0.51)	0.5
Delta LSM/month	9	0.40 (0.23, 0.67)	0.37 (0.24, 0.70)	0.40 (0.23, 0.66)	> 0.9
CSPH regression	9				0.078
Yes: FU‐LSM < 10 kPa		49 (39%)	3 (30%)	46 (39%)	
Yes: ≥ 20% decrease and FU‐LSM 10–19.9 kPa		38 (30%)	2 (20%)	36 (31%)	
No: FU‐LSM ≥ 20 kPa		23 (18%)	5 (50%)	18 (15%)	
No: < 20% decrease and FU‐LSM 10–19.9 kPa		17 (13%)	0 (0%)	17 (15%)	

Abbreviations: CSPH, Clinically Significant Portal Hypertension; LSM, liver stiffness measurement.

^a^ Median (IQR); *n* (%).

^b^ Wilcoxon rank sum test; Fisher's exact test.

### 3.2 LSM Dynamics

LSM decreased after SVR in the overall cohort. The first post‐SVR measurement was performed at a median of approximately 1 year after treatment. No significant differences were observed between patients with and without liver‐related events in terms of absolute LSM change, relative LSM change or rate of LSM decline over time. Similar to LSM at baseline, FU‐LSM was higher in patients who developed liver‐related events (*p* = 0.043); however, there was no significant difference in Delta‐LSM (*p* = 0.7), Delta‐LSM in percent (*p* = 0.5), and Delta‐LSM per month of treatment (*p* > 0.9) as summarized in Table 2.

In Table S1 characteristics of the study population are reported according to changes in liver stiffness during follow‐up.

The Baveno VII criteria for CSPH regression were not significantly different between the two groups of patients (*p* = 0.078). Overall, LSM regression after viral eradication was not discriminative for subsequent clinical outcomes.

### 3.3 Clinical Outcomes

During a median follow‐up of 60 months, liver‐related events occurred in 8% of patients. Ascites and hepatic encephalopathy were the most frequent events, followed by variceal bleeding, portal vein thrombosis, and hepatocellular carcinoma. All decompensation events that occurred during follow‐up are detailed as liver‐related events in the Table S2.

Overall mortality was low, with liver‐related causes accounting for approximately half of deaths. Patients who developed liver‐related events had a higher burden of advanced liver disease at baseline.

### 3.4 Predictive Analysis of Prognostic Markers

Post‐SVR liver stiffness (FU‐LSM) was significantly prognostic for liver‐related events within the first 36 months of follow‐up.

Based on the point estimates of the tAUC, LSM at the first FU post‐therapy showed significant prognostic associations to liver‐related events within the first 36 months after treatment, regardless of the missing data handling strategy (Table 3). The lower limit of the 95% CI of the tAUC stayed well above 0.5 both before (tAUC at 2 years: 0.86 [0.75–0.97]; tAUC at 3 years: 0.82 [0.71–0.93]) and after MI (tAUC at 2 years: 0.92 [0.59–0.99]; tAUC at 3 years: 0.93 [0.67–0.99]), suggesting that missing data did not introduce substantial bias under missing at random (MAR) assumptions, though the wide confidence intervals after MI reflect some degree of uncertainty.

**TABLE 3:** Time‐dependent AUC for LSM performance to predict liver‐related events within 5 years.

	Before imputation				After imputation			
	24 months	36 months	48 months	60 months	24 months	36 months	48 months	60 months
Baseline LSM (kPa)	0.8 [0.71–0.9]	0.79 [0.7–0.89]	0.56 [0.35–0.77]	0.42 [0.24–0.6]	0.75 [0.48–0.91]	0.8 [0.55–0.93]	0.68 [0.45–0.84]	0.57 [0.37–0.74]
FU LSM	0.86 [0.75–0.97]	0.82 [0.71–0.93]	0.62 [0.45–0.8]	0.5 [0.32–0.69]	0.92 [0.59–0.99]	0.93 [0.67–0.99]	0.75 [0.47–0.91]	0.67 [0.46–0.83]
Delta LSM	0.67 [0.56–0.78]	0.69 [0.58–0.8]	0.45 [0.26–0.65]	0.34 [0.19–0.5]	0.39 [0.1–0.78]	0.42 [0.16–0.74]	0.41 [0.19–0.66]	0.38 [0.21–0.59]
Delta LSM in %	0.5 [0.38–0.63]	0.54 [0.41–0.67]	0.35 [0.19–0.51]	0.26 [0.14–0.39]	0.3 [0.07–0.7]	0.3 [0.1–0.62]	0.35 [0.15–0.61]	0.34 [0.18–0.54]

Abbreviations: FU LSM, follow‐up Liver Stiffness Measurement; LSM, liver stiffness measurement.

Baseline LSM (kPa) performed similarly well, both before and after MI, with only the post‐imputation CI for the 24‐month event probability falling slightly below 0.5 (tAUC = 0.75 [0.48–0.91]) due to additional uncertainty associated with the imputation procedure.

After 3 years, at both the 4‐ and 5‐year time points, both FU‐LSM and baseline LSM were not clearly superior to random chance in predicting liver related events (Table 3). A detailed account of clinical events observed during the follow‐up period is provided in supplementary Table 2.

The picture was less clear for Delta‐LSM. While the complete cases analysis indicated moderate predictive capacity within the first three years (tAUC at 2 years: 0.67 [0.56–0.78] and tAUC at 3 years: 0.69 [0.58–0.8]), this could not be confirmed after MI (tAUC at 2 years: 0.39 [0.1–0.78]; tAUC at 3 years: 0.42 [0.16–0.74]). MI introduced considerable uncertainty, as reflected in the wide confidence intervals and the lower bound falling below 0.5, suggesting a potential lack of reliability when using Delta‐LSM to predict liver‐related events.

Delta‐LSM as a percentage of baseline LSM did not show predictive ability beyond chance, regardless of time point or missing data handling method.

Similar to LSM, both FIB‐4 and APRI, measured at both baseline or first FU post treatment, showed significant prognostic associations to liver‐related events for the first 36 months (Table S1). The predictive abilities do not appear to stretch beyond this time frame, and, like LSM, the tAUC for Delta values of FIB‐4 and APRI was not clearly better than random chance.

**FIGURE 1:**
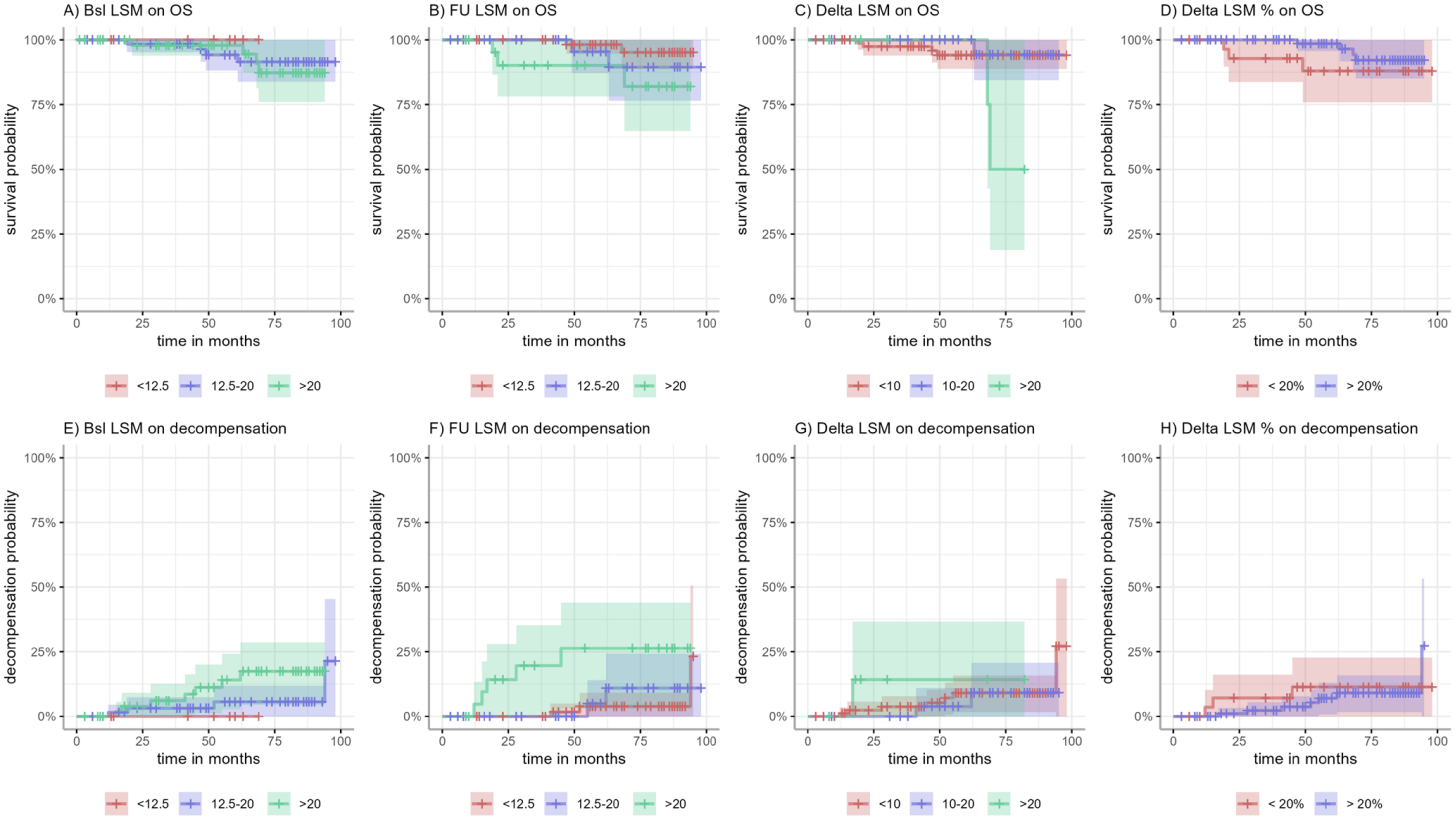
Cumulative incidence curves for heatic decompensation and overall survival in cACLD patients stratified according to baseline LSM (A–E), FU‐LSM (B–F), delta LSM absolute value (C–G) or percentage LSM 20 kPa green line. Delta LSM % 20% blue line.

**FIGURE 2:**
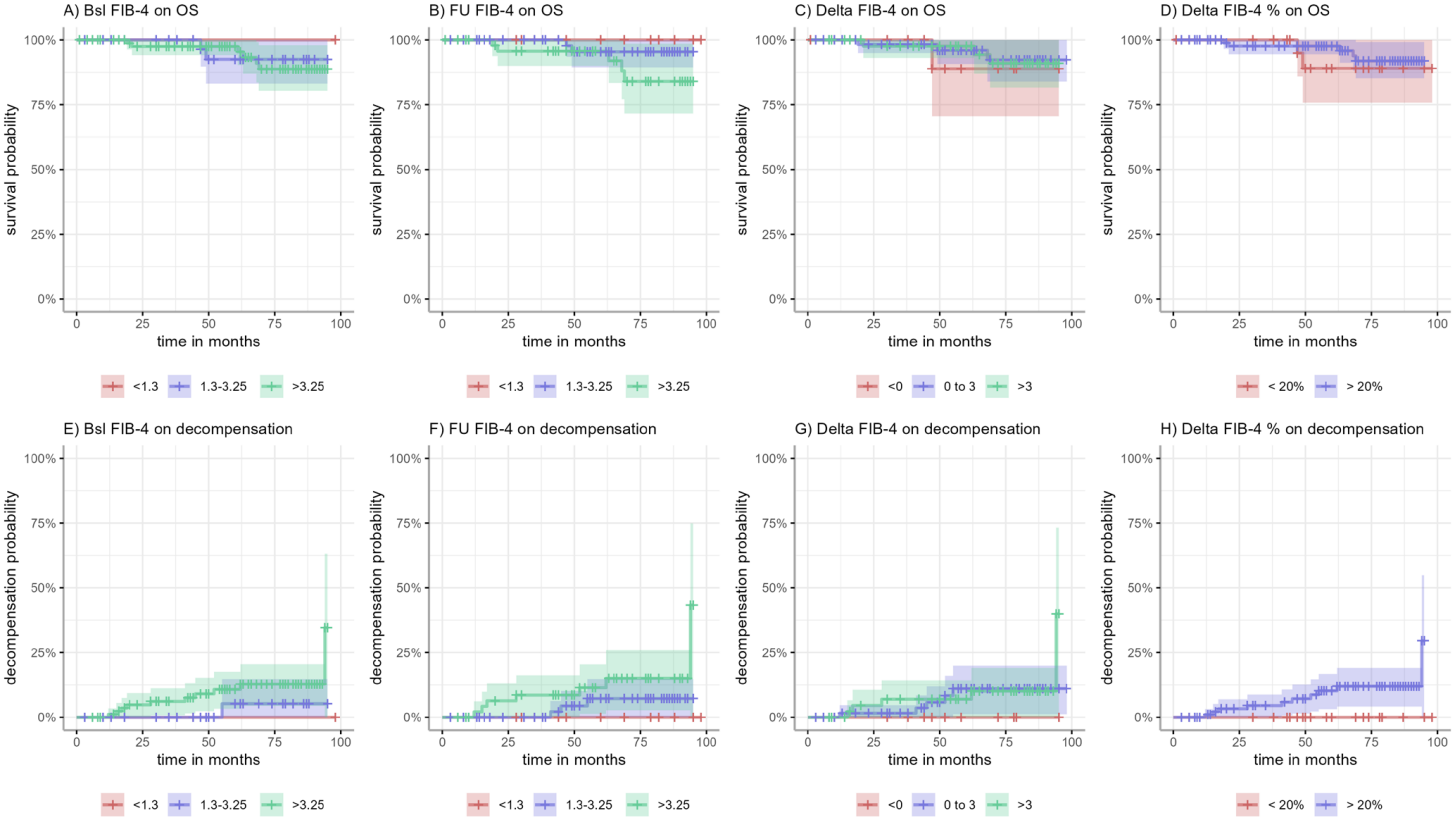
Cumulative incidence curves for hepatic decompensation and overall survival in cACLD patients stratified according to baseline FIB‐4 (A–E), FU‐FIB‐4 (B–F), delta FIB‐4 absolute value (C–G) or percentage (D–H). Value of FIB‐4 3.25 kPa green line. Delta FIB‐4 3 green line Delta FIB‐4% 20% blue line.

All time‐dependent cumulative incidence curves underlying the tAUC calculations can be seen in Figure 1 and Figure 2.

These results are underlined by explorative pairwise comparisons (Table S3). tAUC was not significantly different between Baseline LSM and FU‐LSM at any of the 4 time points (2 years *p* = 0.422, 3 years *p* = 0.504, 4 years *p* = 0.813, 5 years *p* = 0.4), suggesting that both metrics show similar predictive performance. FU‐LSM was significantly superior to Delta LSM (2 years *p* = 0.028, 3 years *p* = 0.007) and Delta LSM % (2 years *p* = 0.002, 3 years *p* < 0.001) at the 2‐ and 3‐year time points, but not thereafter. Like FU‐LSM, baseline LSM was significantly superior to Delta LSM (2 years *p* = 0.022, 3 years *p* = 0.002) and Delta LSM % (2 years *p* = 0.003, 3 years *p* < 0.001) at the 2‐ and 3‐year time points, but not at 4‐ and 5‐years post treatment. There were no significant differences between Delta LSM and Delta LSM%, indicating that both metrics are similarly limited in their predictive ability.

Due to a very limited number of events, we were unable to calculate a single competing risk model encompassing all combinations of FU‐LSM < 10, FU‐LSM > 20 and Delta‐LSM > 20%, as per the Baveno VII criteria. Instead, we fitted three separate univariable models for these criteria as binary predictors.

Only FU‐LSM > 20 was significantly associated with liver related events with SHR = 5.613; *p* = 0.007. Fulfilling the Baveno criteria did not show a significant association with liver related events (SHR = 0.330; *p* = 0.097), indicating that FU‐LSM alone might be preferable in predicting liver related events. The results did not differ by missing data handling strategy. The full results of the competing risk regression analysis can be seen in Table 4.

**TABLE 4:** Univariate competing risk regression analysis on prediction of hepatic decompensation.

Model	Listwise deletion				Multiple imputation			
	*N*	log SHR	SHR	*p*	*N*	log SHR	SHR	*p*
Baveno VII Criteria (Yes)	127	−0.915	0.401	0.150	135	−1.110	0.330	0.097
FU‐LSM < 10	127	−0.571	0.565	0.380	135	−0.626	0.535	0.338
FU‐LSM > 20	127	1.749	5.750	0.006	135	1.725	5.613	0.007
Delta‐LSM > 20%	126	−0.292	0.747	0.680	135	−0.485	0.616	0.635

Abbreviations: FU‐LSM, follow‐up liver stiffness measurement; SHR, subdistribution hazard ratio.

## 4 Discussion

Traditionally, liver disease has been considered a unidirectional process leading to progression from compensated to decompensated liver disease. The availability of effective etiological therapies has revolutionized this paradigm, enabling stabilization or even regression of portal hypertension and liver fibrosis. LSM commonly decreases by removal/suppression of the primary etiologic factor [2, 3, 4].

Baveno VII has introduced simple rules to facilitate the interpretation of changes in LSM in this specific clinical setting. LSM decreases of ≥ 20% in patients with cACLD are defined as clinically significant (i.e., associated with improved outcomes) if LSM is < 20 kPa, or if the value decreases to a LSM < 10 kPa [12].

In our study in 135 patients with cACLD, treated for HCV infection at our center, and follow‐up LSM, we provide evidence that after SVR the absolute value of LSM during follow‐up might be a stronger predictor of liver‐related events than LSM change.

The absolute value of LSM in the follow‐up was higher in patients who developed decompensation during follow‐up (*p* = 0.043), and our findings support that values beyond 20 kPa may be indicative of higher risk for decompensation. On the other hand, we found no significant difference in relative changes of LSM vs. baseline. This confirms recent data suggesting that once current LSM is known, previous values do not add to the prediction of decompensation of liver disease [20, 21].

About 40% of patients who remained compensated in the follow‐up had a FU LSM < 10 (*p* = 0.02). Our results are in line with the Baveno VII criteria for a clinically significant decrease in LSM in identifying patients at substantially reduced risk of liver‐related events.

LSM, as well as FIB‐4 and APRI, measured at both baseline and at the first FU post‐therapy showed significant prognostic associations to liver‐related events within the first 36 months after treatment. The predictive abilities do appear to deteriorate beyond this time frame, and, like for LSM, the tAUC for Delta values of FIB‐4 and APRI was not clearly better than random chance.

The results of the two missing data handling strategies, complete cases and MI, largely agree regarding the estimates of the tAUC. A notable exception to this is that when using complete case analysis, the tAUC of Delta‐LSM appears superior to random chance at 2 years (tAUC = 0.67; 95% CI = [0.56–0.78]) and 3 years (tAUC = 0.69; 95% CI = [0.58–0.8]) post treatment, yet in both cases MI leads to non‐significant results. This indicates that prior results that show significant predictive ability of Delta‐LSM could suffer from bias due to eliminating patients with insufficient follow up from analysis. However, the difference could also stem from the additional variability introduced by the multiple imputation procedure on an already small data set.

In this cohort of patients, we tried to evaluate the Baveno VII criteria for a clinically significant decrease in LSM after HCV cure. Given the small number of liver‐related events observed in the follow‐up, we were unable to calculate a single competing risk model encompassing all combinations of FU‐LSM < 10, FU‐LSM > 20 and Delta‐LSM > 20%, as suggested by the Baveno VII workshop. Instead, we had to fit three separate univariable models for these criteria as binary predictors.

Only FU‐LSM > 20, and not dynamic changes of LSM as compared to the pre‐treatment value, was significantly associated with decompensation, indicating that FU‐LSM alone might be preferable in predicting hepatic decompensation. Thus, in the setting of etiological cure, residual liver disease severity/stage seems to be considerably more important than the magnitude of LSM improvement. Interestingly, similar results have been obtained in studies considering other etiologies of liver disease [21].

Our study has limitations that should be taken into account. It is retrospective, and despite patients were prospectively enrolled in the HCV cohort of our center, we cannot exclude a bias due to lack of measurement of LSM in patients not included in the cohort. Furthermore, we are focusing on a single aetiology, which limits the generalizability of our findings in patients with liver disease due to causes different than HCV.

The number of events observed in the follow‐up is small, which limits statistical power and generalizability, but is in line with other studies [20, 21].

Therefore, the absence of robust evidence for added prognostic value of LSM changes should not be interpreted as evidence of absence of such an effect.

Despite these limitations, our study has several strengths. The cohort was followed for a median of 60 months, and LSM was assessed both before treatment and after SVR using standardized procedures. By evaluating absolute LSM values alongside predefined measures of LSM change, and by reporting both complete‐case and multiple‐imputation analyses, we were able to examine the robustness of the observed prognostic patterns. Across these analyses, post‐SVR LSM showed a more consistent short‐term prognostic signal than change‐based measures, although the limited number of events precludes definitive conclusions regarding incremental prognostic value.

Similarly, FIB‐4 and APRI baseline values were not predictive of outcomes, while values in the follow‐up held prognostic value, confirming that non‐invasive tests, particularly when concordant, can be used in the setting of risk stratification in patients with HCV liver disease achieving SVR. We are also the first to use MI in order to estimate missing data bias due to insufficient follow‐up.

In conclusion, our findings support the EASL and Baveno VII guidelines recommending repeated liver stiffness measurements (LSM) during the follow‐up of patients receiving etiologic treatment for HCV‐related liver disease. Furthermore, our data confirm that the absolute LSM values during follow‐up are the strongest prognostic indicator for predicting a high risk of liver‐related events.

## Author Contributions

Study concept and design (A.B. and F.C.), acquisition of data (F.C., M.R., A.C., C.S. and C.M.), analysis and interpretation of data (J.S., A.B. and F.C.), drafting of the manuscript (F.C., J.S., M.R. and A.B.), critical revision of the manuscript for important intellectual content (all authors).

## Funding

This work was supported by the Data publication Grant from Gilead. The other authors have no disclosures to declare.

## Conflicts of Interest

A.B.: consultant to Boehringer‐Ingelheim; speaker's fee: GE Healthcare; Hologic. F.C.: research grant from Gilead.

## Supporting information


**Figure S1:** Flow diagram for study participants.

**Table S1:** Baseline characteristics of the cohort (*N* = 135) according to LSM decrease.
**Table S2:** Decompensations in the cohort during follow‐up.
**Table S3:** Pairwise comparisons between time‐dependent AUC on liver‐related events.

## Data Availability

Data, analytic methods and study materials will be made available to other researchers upon reasonable request.
