# Supplementary material for: Prognostic Value of Liver Stiffness in Advanced Chronic Liver Disease After Hepatitis C Virus Eradication
Source: Liver Int. 2026 Sep 15;46(10):e70869. doi: 10.1111/liv.70869 (PMC13578848; doi:10.1111/liv.70869)

Supplementary Figure 1 Flow diagram for study participants

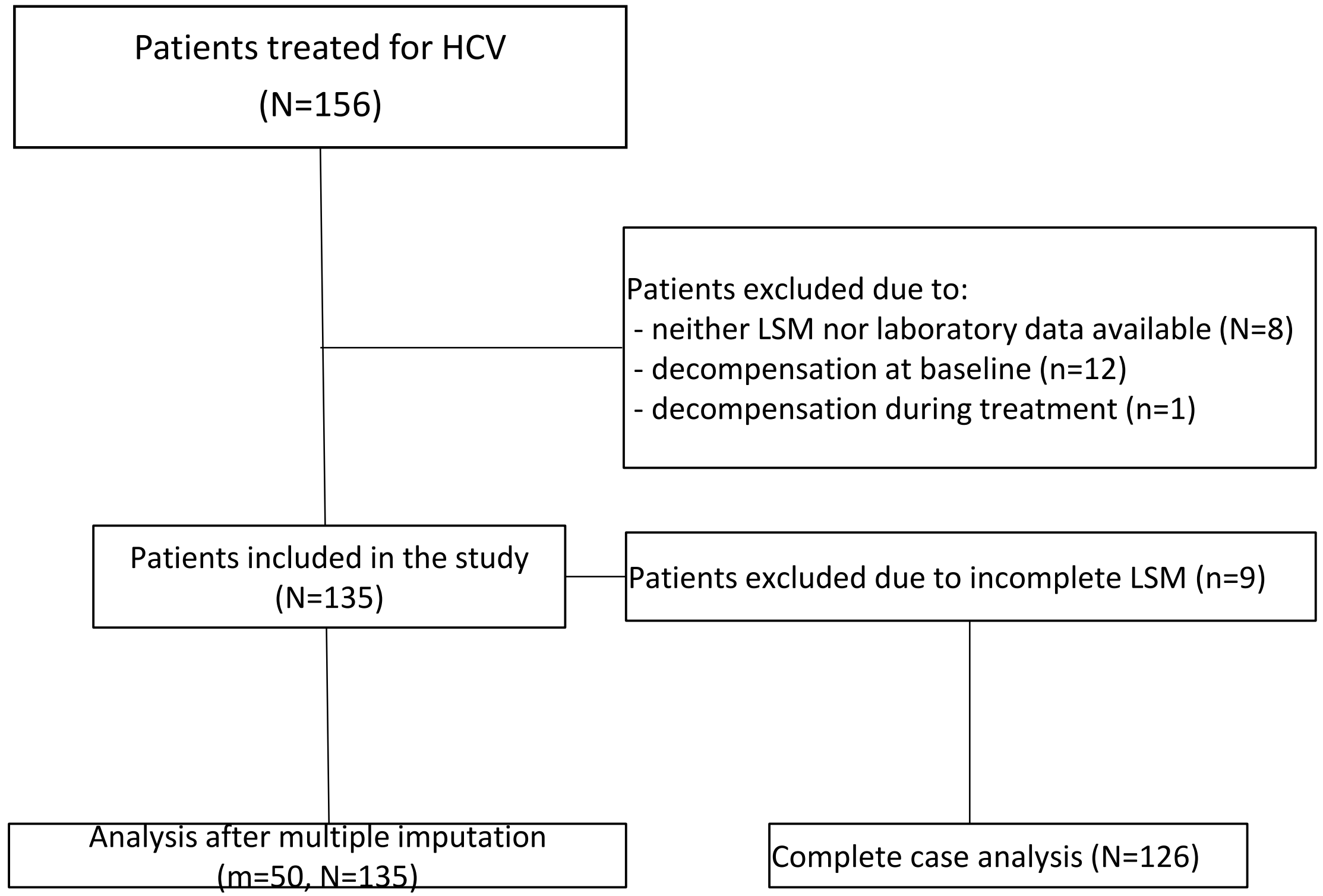

Supplement: Supplementary file 1 [file LIV-46-0-s001.pdf]
