# Supplementary material for: Prognostic Value of Liver Stiffness in Advanced Chronic Liver Disease After Hepatitis C Virus Eradication
Source: Liver Int. 2026 Sep 15;46(10):e70869. doi: 10.1111/liv.70869 (PMC13578848; doi:10.1111/liv.70869)
Supplement: Supplementary file 2 [file LIV-46-0-s002.docx]

Supplementary table 1: Baseline characteristics of the cohort (N=135) according to LSM decrease

| **Characteristic** | **no**, N = 11^1^ | **yes**, N = 115^1^ | **unknown**, N = 9^1^ | **p-value**^2^ |
| --- | --- | --- | --- | --- |
| male sex | 7 (64%) | 62 (54%) | 6 (67%) | 0.7 |
| Age (years) | 53 (48, 69) | 70 (55, 76) | 75 (62, 83) | 0.038 |
| LSM (kPa) | 17 (14, 19) | 18 (15, 25) | 20 (18, 22) | 0.4 |
| LSM groups (kPa) |  |  |  | 0.10 |
| <10 | 1 (9.1%) | 1 (0.9%) | 0 (0%) |  |
| 10-20 | 8 (73%) | 63 (55%) | 3 (38%) |  |
| >20 | 2 (18%) | 51 (44%) | 5 (63%) |  |
| Probe (M o XL) |  |  |  | 0.8 |
| M | 7 (64%) | 83 (72%) | 4 (80%) |  |
| XL | 4 (36%) | 32 (28%) | 1 (20%) |  |
| Fib4 | 2.9 (2.9, 6.0) | 4.9 (2.8, 8.3) | 6.8 (6.2, 12.5) | 0.043 |
| Fib-4>3.25 | 5 (45%) | 80 (70%) | 7 (88%) | 0.2 |
| APRI | 1.24 (0.90, 1.65) | 1.60 (0.85, 2.87) | 2.82 (1.47, 4.07) | 0.094 |
| APRI>2 | 2 (18%) | 49 (43%) | 3 (43%) | 0.3 |
| spleen size (mm) | 130 (120, 160) | 124 (106, 135) | 140 (120, 150) | 0.3 |
| PV diameter (mm) | 13.00 (11.40, 14.00) | 11.00 (10.50, 13.00) | 12.00 (11.00, 13.50) | 0.15 |
| presence and grade of varices |  |  |  | 0.3 |
| 0 (includes previous EBL) | 6 (75%) | 79 (78%) | 5 (56%) |  |
| F1 | 1 (13%) | 17 (17%) | 4 (44%) |  |
| F2 | 1 (13%) | 3 (3.0%) | 0 (0%) |  |
| F3 | 0 (0%) | 2 (2.0%) | 0 (0%) |  |
| Platelet count (×10⁹/L) | 102 (83, 148) | 135 (95, 175) | 84 (80, 106) | 0.057 |
| Albumin (g/dL) | 4.00 (3.90, 4.22) | 4.00 (3.79, 4.30) | 3.50 (3.10, 3.67) | 0.005 |
| Bilirubin (mg/dL) | 0.80 (0.67, 0.87) | 0.89 (0.69, 1.20) | 1.20 (0.82, 2.00) | 0.082 |
| AST (U/L) | 49 (46, 61) | 79 (52, 127) | 122 (55, 162) | 0.016 |
| ALT (U/L) | 57 (30, 84) | 87 (55, 121) | 80 (54, 98) | 0.086 |
| gGT (U/L) | 68 (55, 87) | 73 (45, 131) | 57 (47, 82) | 0.4 |
| INR | 1.10 (1.07, 1.11) | 1.10 (1.05, 1.20) | 1.23 (1.18, 1.29) | 0.012 |
| Glycemia (mg/dL) | 111 (97, 131) | 100 (90, 112) | 92 (84, 101) | 0.15 |
| Creatinine (mg/dL) | 0.63 (0.60, 0.83) | 0.77 (0.68, 0.89) | 0.73 (0.62, 0.88) | 0.5 |
| Child-Pugh class |  |  |  | 0.038 |
| A | 11 (100%) | 104 (93%) | 6 (67%) |  |
| B | 0 (0%) | 8 (7.1%) | 3 (33%) |  |
| C | 0 (0%) | 0 (0%) | 0 (0%) |  |
| MELD | 8.00 (7.00, 9.00) | 8.00 (7.00, 9.00) | 10.00 (9.00, 11.25) | 0.026 |
| body weight (kg) | 73 (68, 95) | 70 (62, 77) | 82 (62, 92) | 0.071 |
| height (cm) | 170 (166, 174) | 167 (160, 173) | 170 (168, 182) | 0.059 |
| BMI | 25.3 (23.9, 29.7) | 24.9 (23.0, 27.1) | 28.7 (22.8, 29.5) | 0.4 |
| BMI>25 | 6 (55%) | 56 (49%) | 5 (56%) | 0.8 |
| BMI>30 | 3 (27%) | 9 (7.8%) | 2 (22%) | 0.045 |
| DMT2 | 4 (36%) | 27 (23%) | 1 (11%) | 0.5 |
| HbA1c (mmol/mol) | 64 (64, 64) | 41 (37, 54) | NA (NA, NA) | 0.13 |
| Dyslipidemia (0 no 1 yes) | 3 (27%) | 26 (23%) | 1 (11%) | 0.8 |
| Statins | 1 (9.1%) | 7 (6.1%) | 1 (11%) | 0.4 |
| Arterial Hypertension | 4 (36%) | 59 (51%) | 5 (56%) | 0.7 |
| Alcohol consumption  (≥20 g/day for women;  ≥30 g/day for men) | 2 (18%) | 14 (12%) | 2 (22%) | 0.4 |
| decompensation |  |  |  | 0.7 |
| decompensation | 1 (9.1%) | 9 (7.8%) | 1 (11%) |  |
| no decompensation | 10 (91%) | 106 (92%) | 8 (89%) |  |
| HCV Genotype 1 | 5 (45%) | 74 (64%) | 5 (56%) | 0.4 |
| HCV Genotype 2 | 2 (18%) | 24 (21%) | 3 (33%) | 0.6 |
| HCV Genotype 3 | 3 (27%) | 8 (7.0%) | 0 (0%) | 0.092 |
| HCV Genotype 4 | 2 (18%) | 11 (9.6%) | 1 (11%) | 0.5 |
| ^1^n (%); Median (IQR) ^2^Fisher's exact test; Kruskal-Wallis rank sum test | | | | |

LSM, liver stiffness measurement; FIB-4, Fibrosis-4 index; APRI, aspartate aminotransferase to platelet ratio index; EBL, endoscopic band ligation; BMI, Body Mass Index; HCV, hepatitis C Virus; PV, portal vein diameter (mm); BMI, body mass index (kg/m²); AST, aspartate aminotransferase (U/L); ALT, alanine aminotransferase (U/L); gGT, gamma-glutamyl transferase (U/L); INR, international normalized ratio; AFP, alpha-fetoprotein (ng/mL); MELD, Model for End-Stage Liver Disease score.DMT2, type 2 diabetes mellitus; HbA1c, glycated hemoglobin

Supplementary table 2: Decompensations in the cohort during follow-up

| outcome | 1 year | 2 years | 3 years | 4 years | 5 years | 8 years |
| --- | --- | --- | --- | --- | --- | --- |
| FU | 125 (92.59%) | 113 (83.70%) | 104 (77.04%) | 87 (64.44%) | 66 (48.89%) | 1 (0.74%) |
| survival | 0 (0.00%) | 2 (1.70%) | 2 (1.70%) | 3 (2.76%) | 4 (3.88%) | 8 (10.05%) |
| decompensation | 1 (0.80%) | 4 (3.28%) | 5 (4.15%) | 7 (6.19%) | 9 (8.71%) | 11 (23.04%) |
| HCC | 2 (1.57%) | 2 (1.57%) | 5 (4.33%) | 9 (8.50%) | 11 (10.98%) | 14 (21.06%) |
| EHT | 0 (0.00%) | 4 (3.63%) | 5 (4.59%) | 8 (7.77%) | 9 (9.07%) | 9 (9.07%) |
| CV event | 2 (1.52%) | 4 (3.17%) | 4 (3.17%) | 4 (3.17%) | 4 (3.17%) | 4 (3.17%) |
| PVT | 1 (0.76%) | 1 (0.76%) | 1 (0.76%) | 2 (1.76%) | 2 (1.76%) | 2 (1.76%) |
| ascites | 1 (0.80%) | 4 (3.28%) | 4 (3.28%) | 5 (4.34%) | 7 (6.88%) | 8 (8.38%) |
| variceal bleeding | 0 (0.00%) | 0 (0.00%) | 0 (0.00%) | 1 (1.01%) | 1 (1.01%) | 1 (1.01%) |
| Hepatic encepalopathy | 0 (0.00%) | 0 (0.00%) | 2 (1.77%) | 2 (1.77%) | 3 (2.94%) | 5 (18.24%) |
|  | | | | | | |
| First line: n and % of patients still under observation, lines 2 to 10: n and % are event rates, n with event is always cumulative, the total number of events until that time. All percentages are cause-specific. FU, follow-up; HCC, hepatocellular carcinoma; EHT extrahepatic malignancy; CV event, cardiovascular events; PVT, portal vein thrombosis | | | | | | |
|  | | | | | | |

Supplementary table 3: Pairwise comparisons between time-dependent AUC on liver-related events.

|  | months since therapy | FU LSM | Delta LSM | Delta LSM in % | Baseline Fib4 | FU FIB-4 | Delta FIB-4 | Delta FIB-4 in % | Baseline APRI | FU APRI | Delta APRI | Delta APRI in % |
| --- | --- | --- | --- | --- | --- | --- | --- | --- | --- | --- | --- | --- |
| Baseline LSM (kPa) | 24 m | 0.422 | 0.022* | 0.003* | 0.91 | 0.691 | 0.935 | 0.174 | 0.941 | 0.999 | 0.551 | 0.052 |
|  | 36 m | 0.504 | 0.002* | <0.001* | 1 | 0.986 | 0.623 | 0.037* | 0.682 | 1 | 0.243 | 0.004* |
|  | 48 m | 0.813 | 0.101 | 0.083 | 0.464 | 0.998 | 0.069 | <0.001* | 0.174 | 1 | 0.009* | <0.001* |
|  | 60 m | 0.4 | 0.184 | 0.212 | 0.296 | 0.994 | 0.042* | 0.003* | 0.127 | 1 | 0.01* | <0.001* |
| FU LSM | 24 m |  | 0.028* | 0.002* | 0.865 | 0.999 | 0.249 | 0.007* | 0.278 | 0.723 | 0.183 | 0.024* |
|  | 36 m |  | 0.007* | <0.001* | 0.56 | 0.865 | 0.109 | 0.001* | 0.142 | 0.538 | 0.1 | 0.003* |
|  | 48 m |  | 0.213 | 0.161 | 0.204 | 0.739 | 0.055 | 0.003* | 0.165 | 0.978 | 0.052 | 0.003* |
|  | 60 m |  | 0.156 | 0.139 | 0.066 | 0.634 | 0.006* | <0.001* | 0.028* | 0.908 | 0.004* | <0.001* |
| Delta LSM | 24 m |  |  | 0.265 | 0.063 | 0.027* | 0.33 | 0.844 | 0.214 | 0.181 | 0.347 | 0.895 |
|  | 36 m |  |  | 0.08 | 0.043* | 0.026* | 0.25 | 0.807 | 0.124 | 0.087 | 0.216 | 0.944 |
|  | 48 m |  |  | 0.441 | 0.613 | 0.233 | 0.868 | 0.994 | 0.692 | 0.09 | 0.939 | 0.981 |
|  | 60 m |  |  | 0.738 | 0.976 | 0.528 | 1 | 0.673 | 1 | 0.334 | 0.949 | 0.5 |
| Delta LSM  in % | 24 m |  |  |  | 0.003* | <0.001* | 0.102 | 0.532 | 0.028* | 0.024* | 0.085 | 0.562 |
|  | 36 m |  |  |  | <0.001* | <0.001* | 0.027* | 0.299 | 0.003* | 0.002* | 0.015* | 0.4 |
|  | 48 m |  |  |  | 0.441 | 0.137 | 0.704 | 0.997 | 0.483 | 0.036* | 0.77 | 0.999 |
|  | 60 m |  |  |  | 0.833 | 0.286 | 0.989 | 0.936 | 0.972 | 0.136 | 1 | 0.799 |
| Baseline Fib4 | 24 m |  |  |  |  | 0.497 | 0.358 | 0.009* | 0.011* | 0.918 | 0.063 | 0.011* |
|  | 36 m |  |  |  |  | 0.785 | 0.345 | 0.006* | 0.075 | 0.993 | 0.132 | 0.003* |
|  | 48 m |  |  |  |  | 0.098 | 0.496 | 0.052 | 0.673 | 0.641 | 0.17 | 0.066 |
|  | 60 m |  |  |  |  | 0.04* | 0.609 | 0.177 | 0.55 | 0.305 | 0.126 | 0.068 |
| FU FIB-4 | 24 m |  |  |  |  |  | 0.265 | 0.014* | 0.034* | 0.185 | 0.08 | 0.022* |
|  | 36 m |  |  |  |  |  | 0.361 | 0.024* | 0.165 | 0.72 | 0.196 | 0.012* |
|  | 48 m |  |  |  |  |  | 0.107 | 0.008* | 0.04* | 0.988 | 0.027* | 0.009* |
|  | 60 m |  |  |  |  |  | 0.14 | 0.041* | 0.018* | 0.925 | 0.02* | 0.013* |
| Delta FIB-4 | 24 m |  |  |  |  |  |  | 0.005* | 1 | 0.91 | 0.869 | 0.129 |
|  | 36 m |  |  |  |  |  |  | 0.004* | 0.997 | 0.805 | 0.929 | 0.044* |
|  | 48 m |  |  |  |  |  |  | 0.022* | 0.98 | 0.404 | 0.817 | 0.112 |
|  | 60 m |  |  |  |  |  |  | 0.111 | 0.999 | 0.224 | 0.636 | 0.064 |
| Delta FIB-4 in % | 24 m |  |  |  |  |  |  |  | 0.245 | 0.265 | 0.592 | 0.976 |
|  | 36 m |  |  |  |  |  |  |  | 0.13 | 0.137 | 0.402 | 0.824 |
|  | 48 m |  |  |  |  |  |  |  | 0.132 | 0.073 | 0.375 | 0.984 |
|  | 60 m |  |  |  |  |  |  |  | 0.39 | 0.057 | 0.628 | 0.772 |
| Baseline APRI | 24 m |  |  |  |  |  |  |  |  | 0.822 | 0.41 | 0.068 |
|  | 36 m |  |  |  |  |  |  |  |  | 0.727 | 0.445 | 0.011* |
|  | 48 m |  |  |  |  |  |  |  |  | 0.224 | 0.234 | 0.106 |
|  | 60 m |  |  |  |  |  |  |  |  | 0.049* | 0.334 | 0.151 |
| FU APRI | 24 m |  |  |  |  |  |  |  |  |  | 0.687 | 0.314 |
|  | 36 m |  |  |  |  |  |  |  |  |  | 0.605 | 0.13 |
|  | 48 m |  |  |  |  |  |  |  |  |  | 0.195 | 0.074 |
|  | 60 m |  |  |  |  |  |  |  |  |  | 0.062 | 0.029* |
| Delta APRI | 24 m |  |  |  |  |  |  |  |  |  |  | 0.065 |
|  | 36 m |  |  |  |  |  |  |  |  |  |  | 0.01* |
|  | 48 m |  |  |  |  |  |  |  |  |  |  | 0.233 |
|  | 60 m |  |  |  |  |  |  |  |  |  |  | 0.22 |

LSM, liver stiffness measurement; APRI, aspartate aminotransferase to platelet ratio index; FIB-4, Fibrosis-4 index
